# Failure in Asthma Control: Reasons and Consequences

**DOI:** 10.1155/2013/549252

**Published:** 2013-12-18

**Authors:** Fulvio Braido

**Affiliations:** Allergy & Respiratory Diseases Clinic, DIMI, University of Genoa, IRCCS AOU San Martino-IST, 16132 Genoa, Italy

## Abstract

Clinical research showed that asthma control is an achievable target. However, real-life observations suggest that a significant proportion of patients suffer from symptoms and report lifestyle limitations with a considerable burden on patient's quality of life. The achievement of asthma control is the result of the interaction among different variables concerning the disease pattern and patients' and physicians' knowledge and behaviour. The failure in asthma control can be considered as the result of the complex interaction among different variables, such as the role of guidelines diffusion and implementation, some disease-related factors (i.e., the presence of common comorbidities in asthma such as gastroesophageal reflux disease (GERD), sleep disturbances and obstructive sleep apnea (OSA), and rhinitis) or patient-related factors (i.e., adherence to treatment, alexithymia, and coping strategies). Asthma control may be reached through a tailored treatment plan taking into account the complexity of factors that contribute to achieve and maintain this objective.

## 1. Introduction 

Asthma is a disease characterized by chronic inflammation of the airways and associated with airway hyperresponsiveness resulting in episodes of wheezing, chest tightness, shortness of breath, and cough, particularly at night or in the early morning. Asthma is recognized as a highly prevalent health problem affecting an estimated 300 million people of all ages, ethnic groups, and geographic origins, with an additional 100 million people estimated to be affected by 2025 [1].

Asthma incidence rate has been increasing for both male and female adults over time, with higher estimates for women [2]. A review of studies published between 1974 and 2004 reported the incidence of asthma in adults as 3.6 and 4.6 asthma cases per 1000 person-years for men and women, respectively [2]. There is now evidence of a plateau in asthma incidence in pediatric population. The Centres for Disease Control and Prevention documented that although asthma prevalence in childhood increased from 3.6% in 1980 to 7.5% in 1997, the lifetime, current symptom, and asthma attack prevalence remained stable between 1997 and 2007, revealing a plateau [3]. Most recent studies have demonstrated similar results both for adults and for children [4] from different European countries.

The presence of asthma is associated with a significant socioeconomic burden [1, 5] due to both direct (such as hospital care, visits, and medications) and indirect costs (such as time lost from work and premature death). Recently, the costs of persistent asthma have been estimated as EUR 19.3-billion in the whole European population aged from 15 to 64 years, with a mean total cost per patient ranging from EUR 509 in controlled asthma up to EUR 2,281 in uncontrolled asthma [6]. Moreover, asthma exerts a considerable social impact not only because it is highly prevalent in many parts of world but also because its presence interferes significantly with many aspects of daily life [7]. Patients with asthma are bothered by the symptoms (in particular cough, shortness of breath, chest tightness, and wheezing) and report considerable impairment in physical activities (such as sports, going up stairs, and shopping). They may have difficulty getting a good night's sleep and may be limited in their work and social life. In addition, the burden of illness has emotional aspects that is, increased levels of anxiety and depression, fear and so on [8]. Guidelines for asthma diagnosis and management have previously focused on the assessment and classification of symptom severity, airflow limitation, and lung function variability [9–12]. Evidence now suggests that asthma severity is not an invariable feature of a patient's condition, but may change with time [13], and it is likely to lead to underestimation of severity, inappropriate therapy, and increased morbidity. Discordance between asthma severity and symptoms/lung function and between severity, inhaled corticosteroid (ICS) and use of reliever medication [13] suggest that classification and treatment of asthma based on severity alone result as inadequate. On the basis of these considerations, after being revised in 2006, the asthma management guidelines issued by the Global Initiative for Asthma (GINA) proposed a new classification of asthma based on the level of control rather than the severity of the underlying disease process [14] and provided a working schema to formalize the classification of disease control. Asthma is defined as “controlled” if the patient reports symptoms and the use of reliever medications twice per week or less, no night waking, no activity limitation or airway obstruction, and no exacerbations; “partly controlled” when symptoms or reliever use are present more than twice per week, and night waking, activity limitation, airway obstruction or exacerbations are present in any week, and “uncontrolled” with the presence of any three or more of these individual features within any week.

The role of the healthcare professional is to establish each patient's current level of treatment and control and then to adjust treatment to reach and maintain control.

More recently, however, an additional concept has been added to indicate that overall asthma control consists of two different components. One is achieving current clinical control or extent of impairment. It refers to the frequency and intensity of symptoms and functional limitations that a patient experiences or has recently experienced as a consequence of asthma and includes measures of day and night symptoms, use of reliever therapy, activity limitations, and lung function. The period for which current clinical control should be assessed is proposed to be the previous 2 to 4 weeks for adults and at least 4 weeks for children. The second component of asthma control is to minimize future risk to the patient by ensuring the absence of asthma exacerbations, the prevention of accelerated decline in lung function over time, and absence of side effects due to medications [15, 16]. These dual components have been particularly emphasized in the recently published Expert Panel Report 3 (EPR3) of the National Heart, Lung, and Blood Institute [16], in terms of “current impairment” and “future risk.”

Several studies have demonstrated that the use of healthcare resources, the level of lifestyle impairment, and quality of life are all strictly linked to the level of asthma control: the better the control, the less impairment, the lower the use of healthcare resources, and the higher the quality of life [17–25]. A 2-year study that evaluated approximately 4000 patients with difficult-to-treat or severe asthma revealed that patients with uncontrolled asthma reported a higher annual mean number of work days lost (7.1 versus 0.4), school days lost (9.1 versus 0.1), and physician visits (5.6 versus 2.4), compared with patients who had controlled asthma [22]. Furthermore, the costs related to asthma management were directly related to the level of control; costs for uncontrolled patients were more than twice the costs of controlled patients (USD 14,212 versus USD 6452; *P* < 0.0001). These results have been confirmed in a recent report by Chapman et al. in approximately 10,500 patients presenting in general practices in Canada [18]; poor daily control was associated with more hospitalizations, emergency and unscheduled visits, and other healthcare contacts.

Moreover, uncontrolled asthma has a considerable impact on patients' ability to function in life activities. Compared with patients with controlled asthma, those with uncontrolled asthma were at higher risk for limitations in outdoor (OR, 2.58) or physical activity (OR, 2.62), and a 66% increased risk of daily activity limitations [26].

## 2. Comparison of Different Asthma Control Tools

The correlations between the international guidelines by GINA and the questionnaires' rating of asthma control has been explored in several studies, highlighting some discrepancies.

A study by Sastre and coworkers [27] confirmed that ACQ is a precise tool in a real-life setting and reveals differences in cut-off points used by three versions of the questionnaire. The optimal cutoff to distinguish between patients with well-controlled asthma and those in whom asthma is uncontrolled was a score of 1.14; lower than the score suggested by Juniper.

A retrospective analysis of O'Byrne et al. [28] compared asthma control as assessed by the Asthma Control Questionnaire (5-item version; ACQ-5), GINA guidelines, or Gaining Optimal Asthma Control (GOAL) study criteria in a large population.

The GINA and GOAL criteria identified a similar proportion of patients within each asthma control classification. The GINA and GOAL control strata were similar in terms of ACQ scores. This analysis showed that the percentages of patients considered by GINA criteria to have controlled and partly controlled asthma and by GOAL criteria to have totally controlled and well-controlled asthma were comparable to an ACQ score of 1.00.

Similar results were found comparing GOAL criteria with the ACQ [29]. For all the ACQ versions, the crossover point between well controlled and not well controlled is close to 1.00. More precisely, if a patient reports an ACQ score of 0.75 or less, there is an 85% chance that his asthma is well-controlled and if a patient has an ACQ score of 1.50 or greater, there is an 88% chance that his asthma is not well-controlled.

A cross-sectional survey was conducted to evaluate whether the ACT can predict GINA defined asthma control, with particular emphasis on the binary split between GINA-defined “partly controlled”/“uncontrolled” asthma versus “controlled” asthma. Almost 3000 patients attending primary care physicians and specialists were recruited in France, Germany, Italy, Spain, the UK, and the USA [30]. An ACT score of <19 correctly predicted GINA “partly controlled” or “uncontrolled” asthma 94% of the time overall and >93% of the time in each country and ACT score >20 predicted GINA-defined controlled asthma 51% of the time.

Another study investigated three different guideline-based tools (GINA, the National Asthma Education and Prevention Program—NAEPP—and the Joint Task Force Practice Parameter—JTFPP) against the ACQ and ACT. Despite having 4 of 5 tested domains in common, the ACQ and ACT showed only moderate agreement with each other. The high level of agreement among the NAEPP, the JTFPP, and GINA should be expected given the similarity in definitions of controlled asthma. The agreement between the ACQ or ACT and the other clinical tools was fair [31].

Recently, Miedinger and coworkers [32] found that an ACT cut-off score of ≤17 best identified uncontrolled asthma according to GINA guidelines.

## 3. Asthma Control Assessment: Validated Tools

Asthma control assessment is of particular importance because, according to current asthma guidelines, treatment should be maintained, stepped-up, or stepped-down based on the basis of the level of control.

Accordingly, the need for tools that can reliably identify and monitor this parameter has become a crucial issue.

Clinicians usually make treatment decisions regarding asthma control on the basis of objective measures of airway status and patient's subjective report of symptoms, use of medications, sleep disturbances, daily limitation, and so forth. They combine these data to estimate the level of asthma control. The use of single clinical or functional parameters seem insufficient to ensure a proper asthma control assessment.

For instance, a study by Boulay and Boulet [33] showed that physicians, evaluating the control level mainly on the basis of symptoms and rescue treatment needs, erroneously identified as “controlled” four out of ten patients who, on the contrary, were “uncontrolled.”

An observational study demonstrated that when physicians estimate the changes in asthma control between two visits on the basis of spirometry and morning and evening Peak Expiratory Flows (PEFs), the clinical judgement may be inappropriate: an unexpected tendency by physicians to overestimate improvement and to underestimate deteriorations in asthma control has been detected [34].

Standardized and validated questionnaires (with or without physiologic measures) to quantify asthma control are now available, including the Asthma Control Questionnaire (ACQ) [35], the Asthma Control Test (ACT) [36], the Asthma Control Scoring System (ACSS) [37], the Asthma Therapy Assessment Questionnaire (ATAQ) [38], and the Perceived Control of Asthma Questionnaire (PCAQ) [39]. These questionnaires are simple and can be easily completed by patients themselves and they make it effortless for clinical practitioners to assess how effectively asthma is controlled.

The ACQ [35] development involved ninety-one asthma clinicians who were members of international asthma guidelines. They identified the seven items in the tool as being the most relevant for determining asthma control. Patients are asked to recall their experiences during the previous week and to answer to the first six questions (nighttime waking, symptoms on waking, activity limitation, shortness of breath, wheeze, and rescue short-acting *β*2-agonist use) on a 7-point Likert scale (0 = no impairment; 6 = maximum impairment). Clinic staff score FEV1% predicted prebronchodilator on a similar 7-point scale. The items are equally weighed and the ACQ score is the mean of the seven items and therefore between 0 (well controlled) and 6 (extremely poorly controlled).

Three shortened versions (symptoms alone, symptoms plus FEV1, and symptoms plus short-acting *β*2 agonists use (SABA) have since also been validated. All four versions of ACQ have demonstrated reliability, validity, and sensitivity to change, with even the smallest change in score of 0.5 considered to be a clinically significant improvement or deterioration in disease control.

Developmental studies have established the cut-off points for controlled asthma (<0.75 points) and not well-controlled asthma (>1.5 points).

The ACT [36] is a patient-completed questionnaire of five items with 5 response options investigating limitations at work or school due to asthma, the presence of daytime or nighttime symptoms, the use of rescue medications, and the subjective perception of the level of asthma control during the previous four weeks. The sum of the scores allows asthma control to be categorized as follows: noncontrolled asthma (5–19 points), controlled asthma (20–24 points), and optimal disease control (25 points). Minimal clinically important differences of 3 has been established.

The ACSS [37] evaluates three types of parameters: clinical (diurnal symptoms, nocturnal symptoms, rescue beta-2-agonists use, activities), physiological (forced expiratory volume in one second (FEV1) and/or peak expiratory flows (PEF) and/or PEF circadian variations), and, as an option, lower airway inflammation (induced sputum eosinophilia). The first section of the questionnaire (clinical parameters) is filled according to the patient's report, on the basis of his/her last week experience, whereas the second and third sections are completed according to the results obtained from additional tests at the time of assessment. The three sections are quantified to obtain a total score of 100% each and a global score is determined using the mean score of the equally weighted sections filled (100% = very well controlled and 0% = not controlled at all). It should be remembered that high symptoms, low pulmonary function, and high eosinophil counts are resulting in a low score.

The ATAQ [38] is a 20-item parent-completed questionnaire for pediatric populations of 5–17 years that generates indicators of potential problems in different categories including symptom control, behavior and attitude barriers, self-efficacy barriers and communication issues.

The PCAQ is an 11-item tool that assesses the patients' perceptions of their ability to manage asthma and its exacerbations. Responses are graded on a 5-point scale, scoring between 11 and 55, with higher scores reflecting greater perceived control of asthma [39].

The C-ACT is a 7-item questionnaire validated in children from 4 to 11 years of age. It evaluates asthma symptoms during day and night, effects of asthma on daily life, and use of rescue medications in the preceding four weeks. The first four questions, each with picture of a face representing child's mood to facilitate comprehension, were answered by the children and the last three by the caregiver in a Likert-type rating scale. The total score ranges from 0 to 27, with higher scores indicating better asthma control. The questionnaire uses a single cut point of a score of 19 to identify children whose asthma is not well controlled.

## 4. The Level of Asthma Control: The Results Emerging from Clinical Trials

Asthma treatment should aim at achieving and maintaining disease control for prolonged periods, with a minimum amount of medications, with due regard to the tolerability of treatment, potential for side effects, and costs. Effective therapies are now available and permit to attain asthma control in the majority of patients as testified by the results of numerous randomized controlled trials.

Aiming at total asthma control by employing strict rules for treatment was the focus of the Gaining Optimal Asthma Control (GOAL) study in 2004 [40], randomized, double-blind, parallel-group study compared the efficacy of fluticasone propionate (FP) and salmeterol/fluticasone propionate combination (SFC) in achieving two composite, guideline-derived measures of control: total control (TC) and well-controlled (WC) asthma. The GOAL [11], defined asthma control based on the treatment goals of the Global Initiative for Asthma/National Institutes of Health guidelines (GINA) [41]. Patients were considered “totally-controlled” if, during the 8 consecutive assessment weeks, they reported no exacerbations, no emergency room attendances, and no medication-related adverse events. “Well-controlled asthma” was achieved if in 7 out of the 8 weeks patients were asymptomatic for at least 5 days per week, and they only required rescue medication during 2 days (or on 4 separate occasions) each week. If any asthma exacerbations, emergency room visits, or medication adverse events occurred during this 8-week period, the patient's asthma control would fail to meet the criteria for control, irrespective of how well their asthma was controlled the rest of the time during the 8 weeks. Beyond revealing the greater efficacy of the combined use of ICS plus long acting *β*-2 agonist (LABA) compared to ICS alone, this study provided important knowledge with regard to asthma management. The goal of total control was achievable in a fair percentage of patients, regardless of disease severity, and increased with treatment time. A posthoc analysis [42] of the Formoterol And Corticosteroid Establishing Therapy (FACET) trial confirmed that in patients with moderate-to-severe asthma, sustained guideline-defined asthma control was possible in a high proportion of patients. All these studies suggest that with the use of the currently available asthma drugs, good control of asthma is a reachable goal in most patients. However, these results obtained in clinical trials where highly motivated and selected patients are carefully followed by a team of researchers, which still need to be translated into real-life practice.

A retrospective analysis of 5 studies comparing Budesonide/Formoterol (BUD/FM) maintenance and reliever therapy versus the 3 maintenance therapies (higher dose ICS, same dose ICS/LAB, and higher dose ICS/LABA plus short-acting b2-agonist) published in 2010 [43] provided several useful insights on the relationship between GINA-defined clinical asthma control and future risk of instability and exacerbations. The main results of the study are the following: the percentage of patients achieving a week of controlled or partly/controlled asthma, increased throughout the study periods, irrespective of the treatment used; ACQ-5 data indicated that control continued to improve over time, irrespective of treatments, with increasing percentages of patients achieving higher levels of control; patients with controlled or partly controlled asthma in any given week had a similar (approximately 75%) estimated probability of maintaining their control level the following week; the probability of having an exacerbation in any week was higher the more uncontrolled the asthma was the previous week; the risk of exacerbations during treatment increased with increasing ACQ-5 cut-point at randomization. The data support the recommendation that having a high level of current control improves stability and reduces the risk of exacerbations while on a regimen that includes an ICS.

A recent randomized, prospective, parallel group study, investigated whether the initiation of maintenance treatment with ICS/LABA alters the time course of asthma outcomes compared to ICS alone. ICS/LABA caused a more rapid improvement in different asthma control measures compared with ICS monotherapy. The mean values of the ACQ score in the BUD/FM group showed an improvement in a shorter period compared to the BUD group. According to the logistic function model, BUD/FM combination significantly improved ACQ, FEV1, FENO, and PD200 at a faster rate than BUD alone over 24 weeks. Especially, the rate of improvement in ACQ with combination therapy was remarkably faster than that with ICS [44].

## 5. The Level of Asthma Control: The Results Emerging from Real Life

Guideline-defined asthma control can be attained and maintained for the majority of patients eligible to participate in a controlled trial setting, but available data indicate us that it is frequently not achieved in real-life practice.

The Asthma Insights and Reality in Europe (AIRE) survey is the first comprehensive, multinational survey assessing the level of asthma control among current asthmatics in Europe [45]. Over 2,800 patients were recruited in seven European countries (France, Germany, Italy, The Netherlands, Spain, Sweden, and UK) and telephone interviews (average duration 25 min) were performed using a structured questionnaire. The results showed that many patients were far from asymptomatic: 46% of patients reported daytime symptoms and 30% asthma-related sleep disturbances at least once a week. Within the last year of the study, 25% underwent unscheduled urgent care visits, 10% had one or more emergency room visits and 7% had overnight hospitalization due to asthma. Only 5.3% of the population surveyed met all the goals of the GINA guidelines.

Similarly, the Asthma Insights and Reality in Asia-Pacific (AIRIAP) assessed whether asthma management met the goals proposed by the GINA guidelines in children and adults and confirmed that also in the Asia-Pacific region [46] asthma control is far from optimal. In a population of 2,323 adults and 884 children, 51.4% of patients reported daytime symptoms, 44.3% had night awakenings due to asthma symptoms during the last four weeks, and 44.7% patient's experienced limitations in physical activity. Moreover, 26.5% of adults and 36.5% of children reported work or school absence in the past year. A high percentage of respondents (43.6%) had been hospitalised or had made unscheduled emergency, unplanned visits due to acute asthma exacerbations during the previous 12 months.

Furthermore, a cross-sectional study performed in 29 countries of America, Europe, and Asia on 7,786 adults and 3,153 children showed that the level of asthma control worldwide falls far short of the goals for long-term management in international guidelines. A significant proportion of patients continue to report symptoms and lifestyle restrictions and to require emergency care. The use of anti-inflammatory preventative medication, even in patients with severe persistent asthma, was low, ranging from 26% in Western Europe to 9% in Japan. Accordingly, the surveys showed that asthma limits the normal activities of a considerable proportion of patients, ranging from 17% in Japan to 68% across Central and Eastern Europe [47].

More recently, the European Community Respiratory Health Survey II found that a major proportion of European adults with asthma were poorly controlled and that the majority of them were receiving suboptimal antiasthma therapy. In more detail, 85% of the asthmatic adults who had used ICSs in the last 12 months were not able to achieve total control of the disease: 49% of them had uncontrolled asthma and 36% had partly controlled asthma. Among those who had not used ICSs in the last 12 months, 18% had uncontrolled and 36% had partly controlled asthma [48].

The International Asthma Patient Insight Research (INSPIRE) study is the first multinational study to focus on patients with a physician-confirmed diagnosis of asthma who were receiving regular maintenance therapy with ICS, with or without an LABA. About 3,400 treated asthma adults were interviewed by phone using a structured questionnaire [49].

Despite 70% of patients being prescribed therapy with ICS plus LABA, only 28% reported well-controlled asthma according to ACQ scores, with 51% of patients classified as having uncontrolled asthma. Most patients (89%) experienced periods of worsening within the last 12 months. Furthermore, although one of the GINA guideline definitions of asthma control is the minimal use of as-needed *β*2-agonists, 74% of patients used their SABA rescue therapy every day in the week preceding the interview. Additionally, 51% of patients required medical intervention for their asthma at least once in the past year, further testifying the poor level of asthma control.

The purpose of the European National Health and Wellness Survey was to evaluate the level of asthma control 10 years after the publication of the GINA guidelines in five European countries: France, Germany, Italy, Spain, and the UK. Nearly 80% of asthma sufferers were treated and 50% of asthma sufferers were “not well controlled.” Among those who were treated, 55% remain not well controlled compared with 95% in the AIRE study. Uncontrolled patients were older, had lower education levels, and were more likely to be obese, depressed, and smokers, when compared to those considered to have controlled asthma. While these results seem to indicate an improvement in the level of control since the AIRE study, they also highlight the disparity between available treatment options and the lack of adequate management [50].

The PRISMA study [51] was designed to include a cross-sectional phase and a 12-month prospective phase with the aim to evaluate the level of asthma control in real life and its evolution during a 1-year followup. A total of 2853 adult patients were recruited in 56 Hospital Respiratory Units in Italy. At baseline 64.4% of patients had controlled asthma, 15.8% partly controlled asthma, and 19.8% were uncontrolled. The number of patients requiring hospitalization or unscheduled visits was lower in controlled (1.8% and 1.6%, resp.) than in partly controlled (5.1% and 11.5%) and uncontrolled patients (6.4% and 18.6%). The main findings from the prospective phase demonstrated that the proportion of patients achieving full asthma control increased over time, reaching 22.2% on the last visit, with 58.7% of patients having controlled asthma, 11.8% partly controlled asthma, and 7.3% were still uncontrolled. The main reasons for lack of asthma control, as declared by physicians, were comorbidities in 36.2% of patients, continued exposure to irritants/triggers in 34.0%, and inadequate adherence to treatment in 27.0%.

The aim of a very recent study involving 462 adult patients in 11 European countries was to evaluate as the economic costs of asthma vary on the basis of disease control [6]. The disease was controlled, partly controlled or uncontrolled in 6.9, 37.9, and 52.2% of the patients, respectively. Controlled patients, compared to partly controlled and uncontrolled, were more often nonsmokers (59.4%), with a later onset of the disease (65.6%) and atopic (72.4%). The mean cost per patient (in the 2010 values) increased as the level of asthma control decreased: 509 Euros for controlled patients, 702 for partly controlled patients and 2281 for uncontrolled ones. After adjusting for the effect of other potential determinants, the lack of disease control resulted as being the strongest predictor of asthma associated costs, which was 3-fold higher among uncontrolled patients in comparison to controlled and partly controlled ones.

A real-life prospective study was recently conducted in Turkey on a populations of 572 adult patients with persistent asthma [52]. The findings revealed an asthma control rate of 61.5% in adult outpatients, which increased upon each follow-up, regardless of the smoking and educational and employment status of the patients. Fixed dose combinations resulted superior in the achievement of asthma control. However, poor asthma control was associated with the incidence of comorbid diseases. The findings have provided valuable data on the positive role of regular monitoring in disease control. The authors therefore advocated regular patient monitoring and patient education to raise awareness and therapeutic expectations, in order to better implement asthma management guidelines and achieve an optimal control of the disease.

A multicentre, retrospective, cross-sectional epidemiologic study enrolled more than 3000 asthma outpatients from 36 general hospitals located in 10 large Chinese cities from April 2007 to March 2008. In total, 28.7% and 45.2% of the respondents had achieved control or partial control of their asthma, respectively, while for 26.2% of patients asthma remained uncontrolled [53]. Discussing the results of this large survey, the authors underlined that despite some improvement that had been achieved in asthma control level, a large gap remained between what had been reached and what was expected to be achieved in developed countries according to the current asthma guidelines. A cross-sectional survey conducted in primary care in UK [54] has pointed out the attention on the tendency of patients to underreport or underestimate their disease severity and overestimate their level of asthma control. The analysis of the survey's results showed that the overall weighted prevalence of uncontrolled asthma in the 2238 enrolled patients was 58%. Surprisingly, 60% of the asthma patients were visiting their physician for a nonrespiratory reason, so it is not strange that 72% of patients with a complaint of respiratory symptoms would have evidence of uncontrolled asthma. However, nearly 50% of patients who presented for a nonrespiratory reason had evidence of uncontrolled asthma. This finding underlined the role of routine evaluation of asthma control for all patients with asthma, irrespective of the reason for the office visit.

## 6. The Relationship between Asthma Control and Health-Related Quality of Life

It is reasonable to hypothesize an association between better asthma control and a reduced burden on health-related quality of life (HRQoL), but few studies have specifically addressed this relationship using validated tools.

### 6.1. Clinical Trials

A retrospective meta-analysis [34] of three randomized double blind placebo controlled studies aimed at evaluating the effect of salmeterol/fluticasone propionate combination therapy on HRQoL of patients with different levels of asthma severity showed that the patients who achieved disease control according to GINA guidelines reported better HRQoL than patients who did not achieve control and indeed these levels were similar to healthy control subjects. Moreover, HRQoL correlated not only with the level of control but also with the treatment allocation: patients treated with salmeterol/fluticasone propionate (SAL/FP) combination therapy reported a significant improvement in HRQoL scores compared to other treatment options, irrespective of the level of control. This suggests that it is possible to reduce the impact of asthma on the patient's life with appropriate drug therapy, even if optimal control is not achieved.

In particular, the correlation between asthma control and HRQoL has been analysed in a cohort of more than 3000 patients involved in the GOAL [17] study. The mean HRQoL scores at the end point were significantly higher in patients achieving total control compared with those achieving good control and also higher in those achieving good control compared to patients who did not achieve control.

The number of patients reporting a clinically significant HRQoL improvement was higher in the group treated with the combination treatment than ICS alone. Moreover, in about half of the patients, when treatment was aimed at achieving total control, HRQoL scores were almost maximal, with the majority of patients having minimal disturbances from their asthma and higher levels of well being.

### 6.2. Real-Life Studies and Surveys

The association between asthma control and HRQoL has also been demonstrated in real-life studies. In a study [55] involving patients with both asthma and rhinitis, only 56% of subjects reached an ACT score ≥20. Patients with controlled asthma showed significantly better HRQoL compared with uncontrolled patients. However, despite HRQoL in asthma improved when patients were controlled, optimal scores were not always seen; that is, the achievement of asthma control did not necessarily equate to the achievement of maximal HRQoL.

The role of rhinitis in determining HRQoL has also been explored. Irrespective of ACT scores and even in patients with controlled asthma, those with additional rhinitis symptoms had a worse HRQoL.

A significant difference between patients with controlled and uncontrolled asthma has been further demonstrated in health status, with worse physical and mental summary scores from the generic SF-12 survey in patients with ACT score ≤19 [56]. The results of these studies have been confirmed in large cohorts including paediatric populations. Recent surveys [57–59] showed that also children with uncontrolled asthma reported significantly lower HRQoL than those who achieved control.

In order to explore the relationship between the perceived asthma control (assessed by PCAQ) and patient's health outcomes, Calfee et al. [60] performed a study on 865 patients. Structured telephone interviews were conducted for patients in a prospective cohort who were observed for hospitalisations due to asthma. Greater perceived control resulted in improved health status and HRQoL.

The EUropean COst of ASthma Treatment (EUCOAST) study was designed to evaluate utilisation of healthcare resources, costs, and HRQL in adult patients with asthma in a primary care setting in France and Spain according to the level of asthma control. The proportion of patients with controlled asthma was significantly higher in France (41%) than in Spain (30%). In both countries, quality of life scores were higher for patients with controlled asthma than patients with partially controlled or uncontrolled asthma [61].

## 7. Reasons for Poor Asthma Control

Though research has shown that good control can be achieved in most patients and valid tools are available to reach this aim, the reality in clinical practice is that asthma remains poorly controlled. That can be considered the result of the complex interaction among different variables (Table 1).

### 7.1. The Role of Guidelines Diffusion and Implementation

Available guidelines for asthma management represent an important and suitable tool aimed at making the entire medical process more effective and efficient: their purpose is to help doctors and patients to make the best decisions about treatment for asthma, by choosing the most appropriate strategies in each specific clinical situation. Despite the effort made to develop and divulge evidence-based guidelines, failure in guidelines implementation remains a sticky issue: about 30–40% of patients do not benefit from a cure program based on scientific evidence, whereas 20–25% of therapeutic choices may be unnecessary and sometimes even harmful [62]. This failure in guidelines implementation has a strong influence on appropriateness of care, clinical efficiency, healthcare costs, and patients' quality of life. It has been underlined that guidelines implementations are complex processes influenced by different factors: some linked to the guidelines themselves (i.e., complexity, credibility, trialability, degree of evidence, concreteness, and transparency), to their implementation (i.e., communication strategies, educational techniques, and use of incentives), and to the sociocultural context (i.e., standard of practice, compatibility of recommendations with the system of existing values in a specific culture system efficiency, and social and clinical norms and habits) represent barriers that limit the achievement of the guidelines' goals and, therefore, the improvement of asthma control.

The need of effective and homogeneous guidelines stimulated the creation of a working group called Grading of Recommendations Assessment, Development, and Evaluation (GRADE) whose activity is endorsed by the WHO. The GRADE method is based on sequential steps that establish the quality of evidence across studies for each important outcome, which outcomes are critical to a decision; the overall quality of evidence across these critical outcomes; the balance between benefits and harms; and the strength of recommendations. Having guidelines structured with the same methodology and evidences gradation could be useful to spread the homogeneous scientific messages. Beyond the evidences of experimental trials on the effectiveness and use reliability, inserting the results of other data that express the relationship of costs benefits and, most of all, the patient values and preferences could make guidelines more applicable in real-life setting [62].

### 7.2. Examples of Disease-Related Factors That Influence Asthma Control

The presence of common comorbidities in asthma such as gastroesophageal reflux disease (GERD), sleep disturbances and obstructive sleep apnea (OSA), and rhinitis affect the airways and can complicate the disease management and the achievement of asthma control.

Epidemiological and pathophysiological evidence indicates that rhinitis and asthma frequently occur as comorbid conditions both in adults and children and that they may be considered as different manifestations of the same inflammatory disease continuum. Approximately 20–60% of patients with rhinitis have clinical asthma, while >80% of patients with allergic asthma suffer from concomitant rhinitis symptoms [63]. Based on the ARIA classification, about one-third of all AR patients present persistent symptoms.

The role of rhinitis must be taken into account in asthma management especially with regard to treatment and prophylaxis. This concept has been clearly demonstrated by Allergic Rhinitis and Its Impact on Asthma (ARIA) [64], and in further clinical trials.

The relationship between rhinitis and asthma control is now well documented in the literature.

A cross-sectional survey in UK general practice showed that out of 3916 asthma patients, 2199 (56%) participants reported mild rhinitis and 924 (24%) severe. Rhinitis patients were 4-5 times more likely to have poorly controlled asthma. Levels of control differed across reported rhinitis groups: the odds of having poor asthma control resulted more than doubled among patients with mild rhinitis and more than quadrupled among patients with severe rhinitis, compared to patients with no rhinitis.

Similarly, a survey on a sample of 253 asthma patients (107 children, 44 adolescents, and 102 adults), indicated that chronic rhinitis was the most important risk factor associated with unscheduled visits due to asthma exacerbations [65].

Both the severity and duration of rhinitis symptoms resulted to be important determinants of asthma control [66]. The odds of having uncontrolled asthma was higher (2.61, *P* < 0.001) in patients with moderate/severe symptoms when compared to those who experienced mild symptoms. The risk of having uncontrolled asthma was significantly higher when the symptoms were persistent rather than intermittent (OR: 1.97, *P* < 0.001).

In a logistic regression models, among patients suffering from severe asthma, moderate/severe rhinitis was a strong predictor of uncontrolled asthma and emergency visits in the year of follow-up. Furthermore, the risk of asthmatic crisis requiring emergency medical assistance or hospitalization was significantly lower in subjects treated for allergic rhinitis compared to those whose allergic rhinitis was not treated [67].

A recent cross-sectional survey, the first nationwide epidemiological study using a standardized validated questionnaire to explore the prevalence of AR in a large population of asthmatic patients (more than 20,000) in China showed that the clinical features of AR had a substantial impact on asthma outcomes. Patients with moderate∖severe AR had a 2.34-fold increased risk of having poorly controlled asthma compared with patients with mild symptoms. AR duration was also associated with poor asthma control. When AR symptoms were persistent, the risk of having poorly controlled asthma was significantly increased [68].

These data support the hypothesis that optimal rhinitis management can lead to the improvement in concomitant asthma and, therefore, a combined treatment strategy should be planned in order to achieve the best possible health status.

### 7.3. The Role of Sleep Disturbances and Obstructive Sleep Apnea

Several studies have explored the link between nocturnal sleep disorders and asthma control, underlining the presence of links and bidirectional influences.

Although nocturnal exacerbations can be related to inadequate asthma control and disturb sleep, poor sleep has been reported also in patients with well-controlled asthma, suggesting that it may be independent from nocturnal asthma. Sleep quality was a significant predictor of asthma control both in severe and not severe asthma [69].

Asthma and obstructive sleep apnea (OSA) are both common disorders [1, 5]. Recent data resulting from cross-sectional and longitudinal studies showed that asthma was associated with both OSA symptoms and diagnoses established by mean of polysomnography [70]. The risk of OSA is approximately doubled in asthmatic populations and asthma severity, female gender, obesity, and gastroesophageal reflux (GER) are important positive moderators of this risk. Conversely, a third of clinical populations with OSA report physician-diagnosed asthma [71]. It has been suggested that obstructive sleep apnea (OSA) is an important contributor to asthma control. Interventional studies focused on small samples demonstrated in patients with asthma and concurrent OSA that the initiation of continuous positive airway pressure (CPAP) therapy result in marked improvement several outcomes, with decreased symptoms, improved peak expiratory flow rate, reduced need for bronchodilator therapy, and resolution of their pattern of nocturnal worsening [72], and improved quality of life [73].

Furthermore, The NAEPP guidelines recommend assessment for OSA in asthma patients with suboptimal control, although evidence contributed only to a grade D (expert panel) recommendation and more research is needed.

A recent study [74] was aimed to determine the association between OSA risk and level of control in asthma. In order to achieve this result, more than 470 patient filled in the Sleep Disorders Questionnaire (SDQ) and the ACT. The univariate association of not well-controlled asthma with high risk of OSAS showed that patients with high risk of OSA had a 3.6 odd ratio to be not well controlled. Obviously, other factors such as obesity, black race, nasal disorders, GERD, and psychiatric diseases are associated with a greater risk of uncontrolled asthma; nevertheless multivariate stepwise logistic regression models of uncontrolled patients showed that, also adjusted for the above-mentioned factors, high OSA risk patients maintain a 2.87 odd ratio to be uncontrolled. In other words, OSA is a potential contributor to overall asthma control on a much larger scale and independent of the other known contributors to asthma control. These results add to the evidence of OSA as a potential contributor to overall asthma control. Similar results had been previously shown by ten Brink and colleagues who introduced OSA among the 13 exogenous and endogenous comorbid factors that influence the level of asthma control [75].

### 7.4. The Role of GERD

A recent prospective study demonstrates a higher incidence of asthma in a general population sample with persistent GERD [76], also after adjusting for BMI. Moreover self-reported GERD is reported in as high as 80% asthmatics, while GERD identified by a positive pH probe occurs at a rate of 38% [77].

GERD may contribute to uncontrolled asthma and may be a potential risk factor for frequent asthma exacerbations and it has been reported more commonly in those with “difficult-to-control” asthma than in patients with well-controlled asthma [78]. In a sample of 226 asthma subjects, a higher percentage of patients without GERD (15.4%) reached good control compared to only 4.5% of those with GERD. On the other hand, more patients with GERD (44.3%) had poor control of their asthma compared to those without GERD (32.3%). Moreover, a higher GERD score was also associated with a lower ACT score [79]. A recent study [80] by means of multivariate logistic regression analyses revealed the independent association of GERD with not well-controlled asthma (odds ratio, 3.12; 95% confidence interval, 1.53–4.88) after other established contributors to asthma control were adjusted. Dixon in a study enrolling 402 asthma patients found that reflux was not associated with measures of asthma control in obese patients [81].

### 7.5. Examples of Patient-Related Factors That Influence Asthma Control

#### 7.5.1. The Role of Adherence

Despite the availability of effective diagnostic tools and safe therapeutic drugs which should allow a satisfactory identification, monitoring, and management of asthma, these alone are not sufficient to obtain disease control. The active involvement of patients in their disease and their therapy and management is essential in reaching and maintaining asthma control. In this perspective the adherence to the treatment plan has become one of the major issues to be faced by medical practice. In the past, the term “compliance” was used to refer to how much a patient's behaviour in terms of drug consumption corresponded to the doctor's prescriptions. This term involves a passive role of the patient viewed as an "executor" of the physician's instructions.

The introduction of the term “adherence” has widened the area of observation: it refers to a collaboration between doctor and patient characterised by a positive sharing of the therapeutic choices and an internalization of the medical prescriptions by the patient [82]. Adherence is enhanced by regular visits to healthcare providers, along with monitoring of symptoms and reemphasis of the avoidance of aggravating factors, changes in lifestyle, and correct management of the therapeutic regimen.

The problem of nonadherence to the therapeutic plan is a serious problem to be addressed in the management of chronic disease, such as asthma.

Nonadherence is reported to range between 20% and 40% in acute illness, 30–60% in chronic diseases, and reaches 50–80% for preventive treatments [83]. The 2003 WHO report on medication adherence [84] quotes the statement by Haynes that “increasing the effectiveness of adherence interventions may have a far greater impact on the health of the population than any improvement in specific medical treatments.”

As with other chronic diseases, a high percentage of asthmatic patients incorrectly follows their doctor's prescriptions, resulting in underdosing, overdosing, or simply incorrect drug usage. Taking into consideration bronchial asthma, adherence to treatment is likely quite low and, according to WHO, it varies from 28 to 43%. The chronic course of a chronic disease such asthma is often characterised by asymptomatic phases or without immediate/short-term risks. These factors contribute to make non-adherence to treatment difficult.

In one of his famous works, Roth and Caron [85] states that “if a patient declares he takes the drugs regularly, often it is not so; if a patient declares that he sometimes forgets some dose, he is usually underestimating the deviation degree from the prescriptions; if a patient says he did not take the drugs, this is usually true.” This picture is probably too negative: according to Roth's perspective; the patient is considered a deceiver who tries to hide his nonfulfilment which separates him from social desirability. According to self-report of treated patients, Roth's vision is justified. The self-report usually provides unreliable data with respect to adherence or non-adherence to treatment. For instance, during a study carried out by Spector on patients suffering from asthma treated with drugs by aerosol [86], adherence was recorded both with an electronic system which registered every inhalation and entries into a personal diary. The results show that 50% of the time patients recorded false consumptions.

Although poor control is the commonest consequence, others include an increase in side effects, disease progression, more complications, the need for emergency visits, the requirement of other diagnostic investigations, and the unnecessary use of stronger drugs, with consequences on the costs of healthcare management.

According to Meichenbaum and Turk [82], nonadherence can be facilitated by the following conditions: presence of chronic diseases, asymptomatic phases of already known pathologies, absence of immediate or short-term risks, recommendation to lifestyle change, therapies whose principal aim is prevention. All these conditions are common to asthma management.

In general, factors connected with non-adherence are gathered in four wider categories [82]: variables connected to the patient, variables connected to the disease, variables connected to the treatment, and variables connected to the physician-patient relationship (Table 2).

Since there are many factors which influence adherence, interventions focused solely on one adherence factor struggle to demonstrate significant benefits. Some subjective factors, such as patient's beliefs, knowledge, and expectations, may play a relevant role in disease management. In particular, patients on long term treatment were sometimes found to have inadequate knowledge about their asthma. A survey [87] exploring the reasons for non-adherence showed that 25% of patients were unaware of the symptoms of asthma; 25% were not taking their medication correctly, 19% were not able to identify worsening signs, and only 40% of patients studied were able to monitor clinical parameters. Although the majority of patients appreciated the necessity for drugs, at least 28% of them were worried about side effects.

It is of great importance that doctors do not underestimate the influence of adherence on achievement of asthma control and other clinical outcomes.

#### 7.5.2. The Role of Disease Perception

Patients' report of symptoms represents an important factor in determining the diagnosis of asthma and influences the treatment plan [88]. However, the self-reported symptoms poorly correlate with pulmonary function measures. This suggests that perception is not necessarily linearly associated with the sensory input, but it depends also on subjective factors.

The proportion of patients who report difficulty in recognizing their own degree of airway obstruction ranges from 15% to 60%, on the basis the measurements used [89]. Poor symptom perception may result in an inadequate management of controller therapy and a consequent overuse of reliever medication. Blunted perception of dyspnoea resulted in related poor health outcomes and increased costs. In a 2-year followup study, patients both with increased and decreased symptom perception exhibited a fourfold-rate of visits to emergency department, a fivefold hospitalization rate and a sixfold rate of near fatal or fatal asthma attacks [90]. These findings demonstrate that perception is a key factor in asthma management.

It is important to remember that the accuracy of symptom perception is not a stable characteristic but depends on a wide variety of cognitive and affective factors. The medical literature suggests that variables such as emotional state, previous life experiences, attributions, contextual information, attentional and learning processes, expectation and prior asthma experiences, personality traits, and psychopathologic disturbances can have an impact on the perception of dyspnea, often independent of airflow obstruction.

Many subjects with asthma learn to associate negative situations and emotional distress with difficult breathing and are thus are likely to overperceive dyspnea. Studies in healthy volunteers and asthma patients show that subjects with high negative emotionality report higher levels of dyspnea than those with low negative emotionality. Similarly, the experimental induction of negative affective states in healthy individuals and patients increases reports of respiratory sensations such as dyspnea. The feeling of unpleasantness related to dyspnea, rather than its sensory intensity, has been related to affective influences [91]. Subjects with asthma may be vulnerable to underperceive dyspnea when experiencing a positive emotional state, potentially reducing the use of prescribed medication [92]. The discrepancy between subjective dyspnea and functional capacity, the effects of inaccurate perception on disease outcomes, and the discordance between a measure of lung function and dyspnea perception should be considered, as this may possibly suggest the need for more careful monitoring.

#### 7.5.3. The Role of Alexithymia

Alexithymia is a psychological characteristic that may play a crucial role in influencing the management of asthma and the achievement of disease control. Interesting suggestions come from the research about alexithymia [93], from the Greek “alexis” (no words) and “thymos” (emotion), a personality trait characterized by difficulty in identifying and describing feelings and distinguishing between feelings and bodily sensations. Individuals with alexithymia have an impaired ability to build mental representations of emotions, and therefore, they misinterpret physical symptoms related to emotions (e.g., tachycardia or tremors) as symptoms of somatic disease. Alexithymia may be considered as a possible risk factor for a variety of medical conditions as it may increase susceptibility to disease development. Various instruments to detect the presence of alexithymia are available; the most widely used is the TAS-20 [94].

Available literature concerning alexithymia and asthma help us to identify the reasons for nonoptimal disease management. Most data are focused on patients with extremely severe or near fatal attacks, suggesting that this psychological characteristic is associated with disease exacerbations. A significantly higher prevalence of alexithymia occurs among patients who have experienced a near fatal asthma attack (36%) compared to patients with matched asthma severity who have not experienced a near fatal attack (13%) [94] or to the general population (5 to 13%). Furthermore, a study that evaluate 270 [94] patients during a severe asthma attack showed that alexithymics reported significantly lower scores for nine symptom/sign categories on the Asthma Symptom Checklist scale. These results may be considered as positive evidence that patients with alexithymia tend to underestimate both physical and emotional components of asthma exacerbations, independent of the severity of the disease. A greater frequency of asthma-induced, recurrent hospitalization has been found in alexithymic patients (37.4% versus 28.4% over 6 months) suggesting that the difficulty in expressing symptom intensity and frequency could lead to the underestimate asthma severity [94]. In addition, a paper by Feldman and colleagues [95] showed that higher alexithymia scores are associated with an increased report of asthma symptoms and with decreased pulmonary function, as indicated by FEV1/FVC%. Finally, the presence of alexithymia has been associated with lower levels of asthma control, poor treatment adherence, and reduced HRQoL scores [96]. In particular, there is a correlation between alexithymia and intentional non-adherence (i.e., the result of an active choice of the patients) [97].

In a real-life study that includes a large sample of asthma patients, the prevalence of alexithymia was 20% compared to 5–13% in the general population, indicating that it is a common feature that characterizes almost 1 patient out of 5 with asthma [98]. Alexithymia did not correlate with disease severity, but it resulted in association with more severe disease experiences and outcomes. In alexithymic patients, asthma is less controlled and independent of the degree of severity and the lack of control leads to a reduced HRQoL. Also the illness perception was different. Alexithymia not only altered the recognition of sensations and symptoms, but also judgements, thoughts, and emotions related to the presence of respiratory allergy. Asthmatic patients with alexithymia reported more serious negative consequences and emotions, affecting physical, psychological, and social aspects of their experiences. Moreover, they tended to perceive and to live asthma as a cyclical disorder, not a chronic condition. Alexithymia seemed also to be associated higher levels of distress.

#### 7.5.4. The Role of Coping Strategies

With the emphasis on self-management of asthma, the role of coping strategies is receiving increasing attention. The study of coping comes from psychological theories of stress management that explore how people deal with stressful events and ongoing situations [99]. The presence of a chronic disease such as asthma constitutes a stress which requires a continuous personal adaptation at different levels: cognitive, emotional, behavioral, and social. Coping efforts may be addressed to modify the situation (e.g., trying to improve asthma control), change the meaning of the experience (e.g., to accept the disease), or mitigate the stress related to the disease management (e.g., to be optimistic about the disease course).

Some researchers have distinguished the coping strategies as problem focused or emotion focused. Problem-focused coping is directed toward managing or modifying a stressful situation or it involves addressing the problem that causes distress. Emotion-focused coping implies efforts to reduce emotional distress caused by the stressful event and to manage or regulate emotions that accompany or result from the stressor.

Active coping strategies, addressed directly to the problem or its meaning, are generally considered to be adaptive, while passive, emotion-focused coping is related to poor adjustment to illness.

Research suggests avoidant coping (ignoring, denying or avoiding a problem) result to be associated with poor HRQoL among adult patients suffering from asthma [96, 100], whereas the use of active coping strategies (taking an active approach to the problem, whether cognitive or behavioural) is associated with higher levels of HRQoL.

It has been shown that asthma patients with effective coping skills tend to experience less psychological morbidity, a feeling of greater personal control over asthma, and better long-term management of the disease [101]. These effective coping skills are characterized by a positive vision of the disease without minimizing its potential danger and by active, cognitive strategies result in flexible and diversified behaviors.

In contrast, the use of avoidance strategies is associated with a lower level of asthma control, with a consequent worsening of clinical outcomes, such as a greater number of hospitalizations, unscheduled health care visits, and episodes of near fatal asthma [102]. Patients with ineffective coping strategies tend to have difficulty treating and managing their asthma [103]. For example, it has been shown that patients identified as having brittle asthma tend to delay seeking care for more than 7 days after a change in symptoms. Asthmatic subjects that use avoidant strategy are less adherent to medical therapy and prone to reliever drug abuse and to inadequate controller drug use.

Modifying coping strategies is more effective than enhanced disease state knowledge in improving asthma treatment outcomes. Coping-skills training, based on social cognitive theory, stresses the use of adaptive coping methods and problem-solving skills. These skills can increase a subjective sense of competence and self-efficacy in dealing with a wide range of daily demands and health issues. Educational interventions aimed at improving coping strategies resulted in effective increasing of the flexibility of the strategies themselves and their functionality, with benefits not only on asthma symptoms but also on psychological functioning, sense of wellbeing and anxiety [104].

A large, cross-sectional survey explored coping in asthma management with the aim to provide a detailed global picture of the issue, investigating the use of coping strategies both from the patient and physician perspectives. A large sample of patients (6474) and general practitioners (3089) was involved [100]. The use of active coping strategies were described in approximately one-half of the patients, while the physicians tend to report a significantly lower frequency of effective coping strategies in their patients. A similar percentage of patients and greater percentage of physicians reported the use of negative strategies, with “rely on fate or faith” chosen more frequently by patients rather than physicians. Age and level of education did not seem to influence the coping style, but females were more prone to employ passive/avoidant behaviors. The degree of concordance between patients and GPs choices was only fair, indicating inadequate patient-physician communication.

### 7.6. Examples of Doctor-Related Factors That Influence Asthma Control

Two recent questionnaire-based studies offered glimpses of links between physicians' behaviour and the doctor patient relationship and how this affects patients' knowledge of their own disease, suggesting important elements that may influence asthma management.

In the first study [105], 811 general practitioners (GPs) and 230 specialists in respiratory medicine, who attended a continuing medical education program, completed a questionnaire on aspects related to the pathogenesis of asthma and its control, the applicability of research and guidelines in daily practice, and the doctor-patient relationship. Barriers to achieving asthma control included the limited level of knowledge among GPs and specialists regarding the use of instruments such as the ACT, which was only used by 20% of GPs and 43% of specialists. When asked to identify the level of asthma control, only 20% provided correct answers. Although over 90% of physicians considered chronic inflammation to be the main characteristic of asthma, up to 40% of patients were considered to not require long-term treatment. Both GPs and specialists preferred fixed dose continuous regimes (58% and 54%, resp.) and believed that a self-management plan was feasible only in a very small percentage of patients. Another noteworthy finding was that neither GPs nor specialists had complete trust in the applicability of clinical trial findings and guidelines in real life.

Regarding the doctor-patient relationship, less than one third of GPs and only a fifth of specialists adopted a cooperative approach (aimed at involving the patient in an active way in the entire treatment process and creating a partnership with him), showing instead a “paternalistic” particularly in the case of GPs. This approach is not ideal for management of a chronic disease such as asthma, since it involves a passive role on the part of the patient with limited autonomy. For both GPs and specialists, the level of patient education was the key determinant of adherence. Patient expectations and treatment peculiarities were considered less important aspects. The findings of this survey provide, on the one hand, an explanation for the poor level of asthma control seen in daily practice, and on the other, evidence for the further need for the development of targeted information and patient education programs.

In a second survey of the same authors [106], GPs involved in asthma medical education programs were asked to answer questions concerning their experience on clinical aspects of asthma management (pathogenesis and control, applicability of research, and guidelines to daily practice) and their doctor patient relationships and patient educational strategies. Each physician recruited 3 asthma patients who each indicated 3 aspects of their disease (from a choice of 10 possible options) that they felt they were less informed about. A total of 2,332 GPs and 7,884 patients participated in the study. Only a half of GPs thought that control could be reached independently of disease severity, and a relevant percentage of physicians minimized their responsibility in unsatisfactory asthma control, declaring that control could be reached only by more scrupulous patients. Most GPs thought that a long-term treatment was applicable in the majority of patients but 1 out 4 believed that this strategy was applicable only in patients with specific characteristics.

Surprisingly, the topic patients chose as feeling least informed about was “the meaning of asthma control” and this choice seems to be significantly associated with physician's behaviour.

These patients were less aware of the meaning of asthma control, when their physicians did not explore how the patient perceived their own disease, failed to provide additional supporting educational material, did not listen to the patient, and did not involve the patient in their ongoing asthma management plan. It is relevant to notice that this topic was ranked lower at 8th place, when including specialists and patient's belonging to patients' associations. This demonstrates that physicians tend to take for granted some important aspects of patient education in asthma management. If patients are not sufficiently educated and do not understand the meaning of control, this facet of doctor patient interaction may be a further barrier to their education and by extension their optimal management.

## 8. Conclusions

Although clinical trials have demonstrated that asthmatic patients may reach an optimal level of disease control, which implies minimal or absent disease impact on patient life, what happens in real life remains far from ideal.

This objective may be reached through a tailored treatment plan taking into account the complexity of factors that contribute to achieve and maintain this objective.

## Figures and Tables

**Table 1:** Factors that influence the achievement and the of asthma control maintenance.

Reasons of poor control	Variables	Examples
Disease-related	Comorbidities	Rhinitis, rhinosinusitis, gastrooesophageal reflux, obstructive sleep apnoea, and obesity
	Triggers	House dust mite, pets, occupational exposure, exercise, drug, passive smoking, new allergens, aspirin, and beta-blockers
	Asthma type	Aspirin-sensitivity, neutrophilic activity, and severe therapy-resistant

Patient related	Sociodemographic factors	Female sex, education below secondary level, adolescence, and elderly age
	Adherence	Undertreatment, overtreatment, irregular visits to healthcare providers, insufficient monitoring of symptoms, and no modifications in lifestyle
	Psychiatric comorbidity	Anxiety and depressive disorders
	Psychological characteristics	Alexithymia (a personality trait characterized by difficulty in identifying and verbally expressing feeling) and inadequate coping strategies
	Perceptions	Tendency to tolerate symptoms, exacerbations and lifestyle limits as an inevitable consequence of asthma
	Expectations	Low expectations and aspirations about the achievable degree of control
	Behaviours	Smoking habits Incorrect use of inhaler leading to ineffective/reduced drug delivery
	Knowledge	Inadequate information about the disease's treatment.

Doctor related	Misdiagnosis	Limited awareness of asthma prevalence inadequate assessment
	Knowledge of current guidelines	Lack of consciousness and familiarity about guidelines availability
	Attitude towards guidelines	Difficulty in accepting a particular document or the concept itself of the guidelines Lack of confidence in personal abilities to put the recommendations into practice Expectations of failure in following guidelines
	Guidelines implementations	Difficulty changing deep-seated routines

**Table 2:** Causes of nonadherence.

Factors linked to the patient	(i) Presence of physical disorders (ii) Cognitive difficulties (iii) Psychiatric comorbidities (iv) Age (children, adolescents, and elderly present high risk of nonadherence) (v) Knowledge (vi) Expectations (vii) Social and family support (viii) Coping style

Variables linked to the disease	(i) Chronicity (ii) Symptom stability (iii) Absence of symptoms

Variables linked to the treatment	(i) High number of daily doses (ii) Presence of side effects (iii) Complexity of the therapeutic regimes (iv) Ease of use (v) Costs

Variables related to the doctor-patient relationship	(i) Bad relationship (ii) Inappropriate doctor or patient behaviour
